# Association of the immunogenicity of intramuscular SARS-CoV-2 mRNA vaccination with computed tomography muscle images in patients with muscular disorders

**DOI:** 10.3389/fimmu.2024.1479321

**Published:** 2024-12-20

**Authors:** Tomoaki Naka, Michinori Funato, Kunihiko Yasuda, Takahiro Nakayama, Satoshi Kuru

**Affiliations:** ^1^ Department of Pediatric Neurology, NHO Nagara Medical Center, Gifu, Japan; ^2^ Department of Pediatric Surgery, NHO Nagara Medical Center, Gifu, Japan; ^3^ Department of Neurology, Division of Neuromuscular Diseases, Yokohama Rosai Hospital, Yokohama, Japan; ^4^ Department of Neurology, NHO Suzuka Hospital, Suzuka, Japan

**Keywords:** COVID-19, muscular disorder, severe motor and intellectual disabilities, anti-SARS-CoV-2 S-RBD IgG, intramuscular SARS-CoV-2 mRNA vaccination, CT value

## Abstract

**Backgrounds:**

Intramuscular mRNA vaccines against severe acute respiratory syndrome coronavirus 2 (SARS-CoV-2) have a low intensity and latency of antibody response in patients with muscular disorders (MDs). However, the mechanisms involved in this phenomenon remain unknown. This study aimed to clarify the mechanism of the low immunogenicity of intramuscular SARS-CoV-2 mRNA vaccination in patients with MDs.

**Methods:**

We evaluated 44 individuals, including 23 patients with MDs and 21 patients without MDs. The median age of the patients was 39 years (range 20–63 years). The anti-SARS-CoV-2 spike protein receptor-binding domain (S-RBD) IgG levels from a previous study were reused. Mean computed tomography (CT) values and areas of the deltoid muscle from CT images were measured, and they were compared with the anti-SARS-CoV-2 S-RBD IgG levels.

**Results:**

One month following the second vaccination, the antibody response among patients with MDs showed a low tendency compared with that among patients without MDs. Surprisingly, a similar pattern was observed when comparing mean CT values. Patients with mean CT values of zero HU and lower had a lower tendency of antibody response after the intramuscular administration of SARS-CoV-2 mRNA vaccines.

**Conclusion:**

The low immunogenicity of intramuscular SARS-CoV-2 mRNA vaccination against MDs may be mainly affected by disease type and MD pathogenesis. However, SARS-CoV-2 immunization in patients with MDs warrants further investigation.

## Introduction

Severe acute respiratory syndrome coronavirus 2 (SARS-CoV-2) mRNA vaccines administered intramuscularly have played a significant role in protecting against severe coronavirus disease-19 (COVID-19) (1, 2). We previously described that the intensity and latency of antibody response to intramuscular SARS-CoV-2 mRNA vaccines were suppressed in patients with muscular disorders (MDs) compared with those in patients without MDs, and we described that MDs may be a key contributor in predicting the antibody response of intramuscular SARS-CoV-2 mRNA vaccines (3). In a previous study, we found correlations between anti-SARS-CoV-2 spike protein receptor-binding domain (S-RBD) IgG antibody levels and patient age, specifically in patients with Duchenne muscular dystrophy (DMD), which causes progressive muscular degeneration and wasting (3). We speculated that both the quality and quantity of the muscle might affect the immunogenicity of intramuscular SARS-CoV-2 mRNA vaccination in patients with MDs, and we hypothesized that SARS-CoV-2 immunization in patients with MDs warrants further investigation (3).

In 1985, Kawai et al. (4) described the usefulness of muscle computed tomography (CT) in patients with DMD. Since then, CT as with magnetic resonance imaging (MRI) are considered the gold standard for the noninvasive assessment of muscle quality and quantity and have been used to evaluate patients with MDs in Japan (5, 6). Although MRI has been used to quantify muscle volume and composition, thus allowing for the differentiation of muscle tissue from adipose, edematous, and fibrous connective tissues (7, 8), CT has the ability to measure muscle quality and quantity, easily and immediately. Therefore, the use of CT for research on muscles is becoming more common (8–10).

In this study, mean CT values and areas of the deltoid muscle were measured using the CT images of patients with or without MDs who were vaccinated with two doses of intramuscular SARS-CoV-2 mRNA vaccines. We discussed the findings on the association of the immunogenicity of intramuscular SARS-CoV-2 mRNA vaccination with CT muscle images in patients with or without MDs.

## Subjects and methods

### Ethical compliance

This study was approved as an additional study on the immunogenicity of intramuscular SARS-CoV-2 mRNA vaccination in patients with or without MDs by the ethics committee of Nagara Medical Center (approval number: ID 2023-10 and 2024-1). This study was assessed using an opt-out consent approach. This means that patients or the parents of patients with intellectual disabilities are included in the retrospective study unless they give their express decision to be excluded. This approach was adopted because of the low risk and potential benefits of the study to patients regarding intramuscular SARS-CoV-2 mRNA vaccination.

### Study participants and evaluations

This study included 75 patients with or without MDs, particularly those with severe motor and intellectual disabilities (SMIDs). Detailed information on the patients and the methods of the previous study were described in a previous article (3). Patients with or without MDs were vaccinated with two doses of BNT162b2 vaccines (Pfizer-BioNTech). Serum samples were collected from each patient on the day of the second dose of vaccination and then after one, three, and six months. Anti-SARS-CoV-2 S-RBD IgG levels were measured using the Abbott SARS-CoV-2 IgG II Quant assay (Abbott, Sligo, Ireland). We subsequently converted the AU/mL to the BAU/mL of the international standard using a conversion factor of 0.142, and analyzed the results.

Moreover, we examined the mean CT values and areas of the deltoid muscle from CT images performed during the same period as the previous study by using image J software (11). We then compared the immunogenicity of intramuscular SARS-CoV-2 mRNA vaccination with the mean CT values or areas of the deltoid muscle.

### Statistical analysis

The statistical significance of the data regarding the levels of anti-SARS-CoV-2 S-RBD IgG antibodies (female vs. male, aged ≤ 31 years vs. > 32 years, bedridden vs. others, with MDs vs. without MDs, mean CT value ≤ 0 HU vs. > 0 HU, and the area of the deltoid muscle ≤ 1000 mm^2^ vs. > 1000 mm^2^) was determined using the paired t-test. The statistical significance was set at *p* < 0.05 for the data analysis. Statistical analyses were performed using EZR (Saitama Medical Center, Jichi Medical University, Saitama, Japan) (12), which is a modified version of R commander for performing statistical functions and is frequently employed in biostatistics (The R Foundation for Statistical Computing, Vienna, Austria).

## Results

### Characteristics of participants

Among the 75 patients in the previous study, 31 were excluded from the current study because they did not undergo CT examination during the previous study period or because the mean CT values and areas of the deltoid muscle from their CT images were not properly evaluated. We analyzed the data of 44 research participants.

Among these 44 patients, 13 had DMD (no patient treated with glucocorticoid steroids), 5 had Fukuyama congenital muscular dystrophy (FCMD), 5 had Myotonic dystrophy type 1 (DM1), 14 had cerebral palsy, 2 had spinocerebellar degeneration, 1 had sequela of brain infarction, 1 had Reye’s syndrome, 1 had Angelman syndrome, 1 had Rett syndrome, and 1 had Miller–Dieker syndrome. This study included 18 females (40.9%) and 26 males (59.1%). The median age of the 44 patients was 39 years (range: 20–63), and the mean age was 40.3 years.

### Anti-SARS-CoV-2 S-RBD IgG levels with respect to each factor

When we analyzed anti-SARS-CoV-2 S-RBD IgG antibodies with respect to sex, age, and ambulation factors, no significant differences were observed between females (18 patients [40.9%]) and males (26 patients [59.1%]), between those aged 31 and under (17 patients [38.6%]) and those over the age of 32 (27 patients [61.4%]), and between bedridden patients (29 patients [65.9%]) and other patients (15 patients, 34.1%) (**Table 1**).

**Table 1:** Comparative results of anti-SARS-CoV-2 S-RBD IgG antibody levels in each characteristic.

Characteristic	Number (%)	Anti-SARS-CoV-2 S-RBD IgG Antibody (BAU/mL) (Mean (SD))							
		Day 0	p-value	1 month	p-value	3 months	p-value	6 months	p-value
Sex									
Female	18 (40.9%)	313.7 (477.3)		1997.2 (1591.2)		496.3 (410.9)		142.7 (108.1)	
Male	26 (59.1%)	191.7 (192.7)	0.525	1725.5 (2592.7)	0.119	526.9 (675.6)	0.556	151.0 (140.3)	0.838
Age (Years)									
≤ 31	17 (38.6%)	348.7 (493.3)		1935.6 (1876.7)		582.6 (659.7)		157.6 (156.1)	
> 32	27 (61.4%)	174.2 (169.7)	0.321	1774.4 (2443.7)	0.615	471.4 (526.1)	0.916	141.4 (107.2)	0.679
Ambulation									
Bedridden	29 (65.9%)	293.3 (404.3)		1771.2 (2349.2)		494.8 (539.1)		145.2 (108.6)	
Others	15 (34.1%)	141.6 (105.7)	0.164	1963.3 (2017.7)	0.947	552.3 (661.2)	0.973	152.4 (160.6)	0.873
Disease/Disorder									
MDs	23 (52.3%)	197.6 (174.1)		1168.5 (1041.2)		375.0 (348.9)		128.0 (94.6)	
Non-MDs	21 (47.7%)	289.8 (458.3)	0.395	2568.5 (2885.5)	0.0701	667.1 (730.6)	0.0794	169.1 (154.3)	0.245
CT value (HU)									
≤ 0	21 (47.7%)	193.1 (173.2)		1080.5 (871.7)		346.5 (297.2)		121.0 (80.9)	
> 0	23 (52.3%)	285.9 (440.3)	0.308	2527.0 (2810.1)	0.0899	667.7 (719.6)	0.178	171.9 (155.5)	0.426
CT area (mm^2^)									
≤ 1000	18 (40.9%)	333.5 (478.0)		1609.1 (1362.9)		491.1 (429.6)		150.9 (103.5)	
> 1000	26 (59.1%)	178.0 (180.9)	0.152	1994.2 (2673.8)	0.603	530.5 (667.4)	0.291	145.3 (142.7)	0.172

SD, standard deviation; MDs, muscular disorders; CT, computed tomography.

*p* -values represent Female vs Male, ≤ 31 vs > 32, Bedridden vs Others, MDs vs Non-MDs, ≤ 0 vs > 0, and ≤ 1000 vs > 1000.

**Figure 1:**
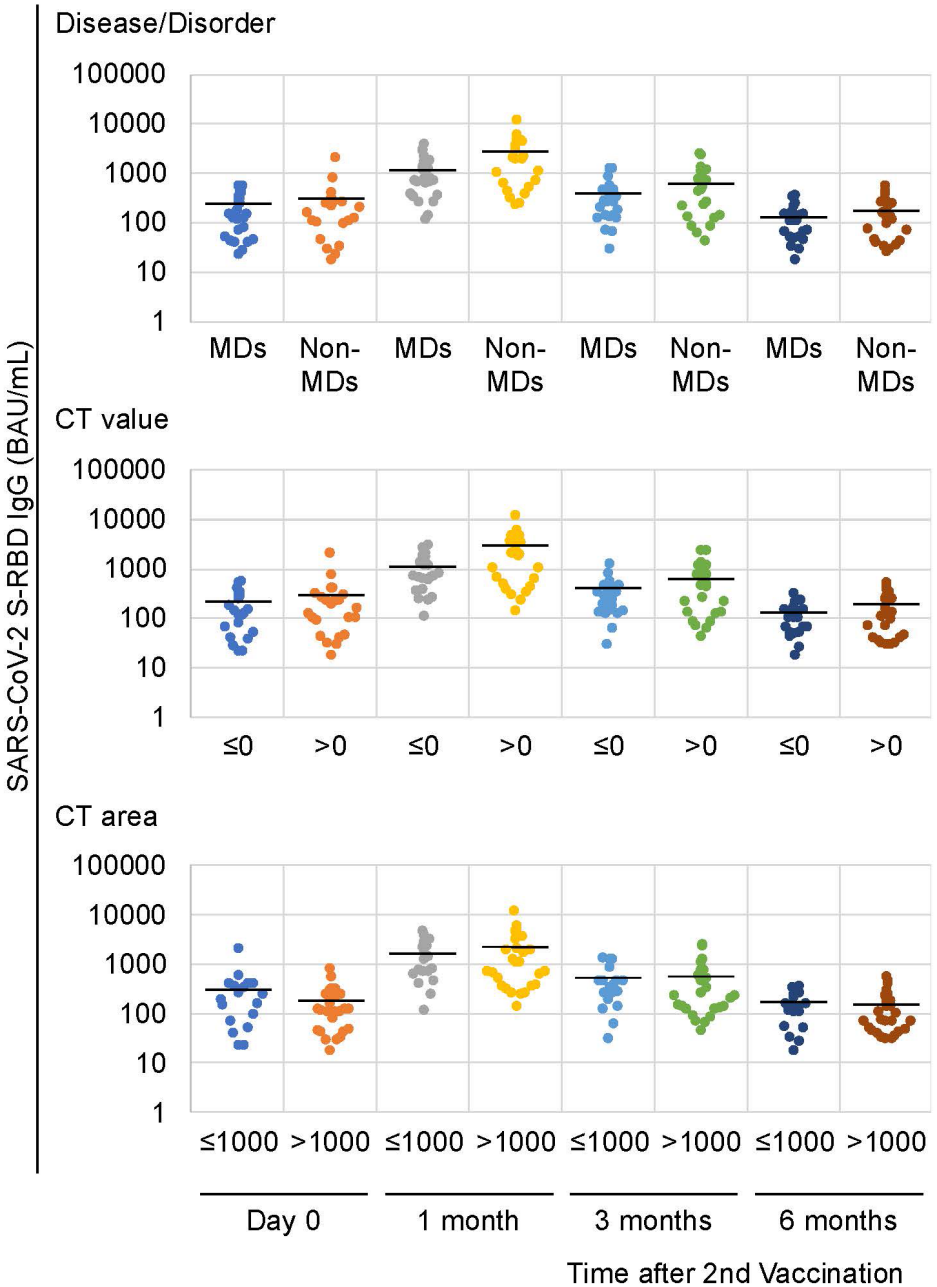
Anti-SARS-CoV-2 S-RBD IgG antibody levels following the second vaccination. Similar patterns were observed in the disease/disorder and the CT value factors 1 month following the second vaccination. Solid lines represent the mean antibody levels in each group.

Then, we divided these patients to MD (including the DMD, FCMD, and DM1) and non-MD (including SMIDs and others) groups. Twenty-three (52.3%) of the 44 patients had MDs, whereas 21 (47.7%) did not have MDs. The patients with and without MDs had median ages of 34 and 48 years, respectively, and their mean ages were 35.0 and 46.0 years, respectively. When we compared the anti-SARS-CoV-2 S-RBD IgG antibodies between patients with and without MDs, patients with MDs (mean 1,168.5 BAU/mL) showed a lower tendency than patients without MDs (mean 2,568.5 BAU/mL) at one month following the second vaccination (*p* = 0.07; **Table 1**; **Figure 1**). Although a similar tendency was also observed three months following the second vaccination (*p* = 0.08), no differences between patients with and without MDs were observed during the first day of the second vaccination followed by six months post vaccination with p = 0.395 and 0.245, respectively (**Table 1**; **Figure 1**).

### Anti-SARS-CoV-2 S-RBD IgG levels with respect to CT imaging

**Figure 2:**
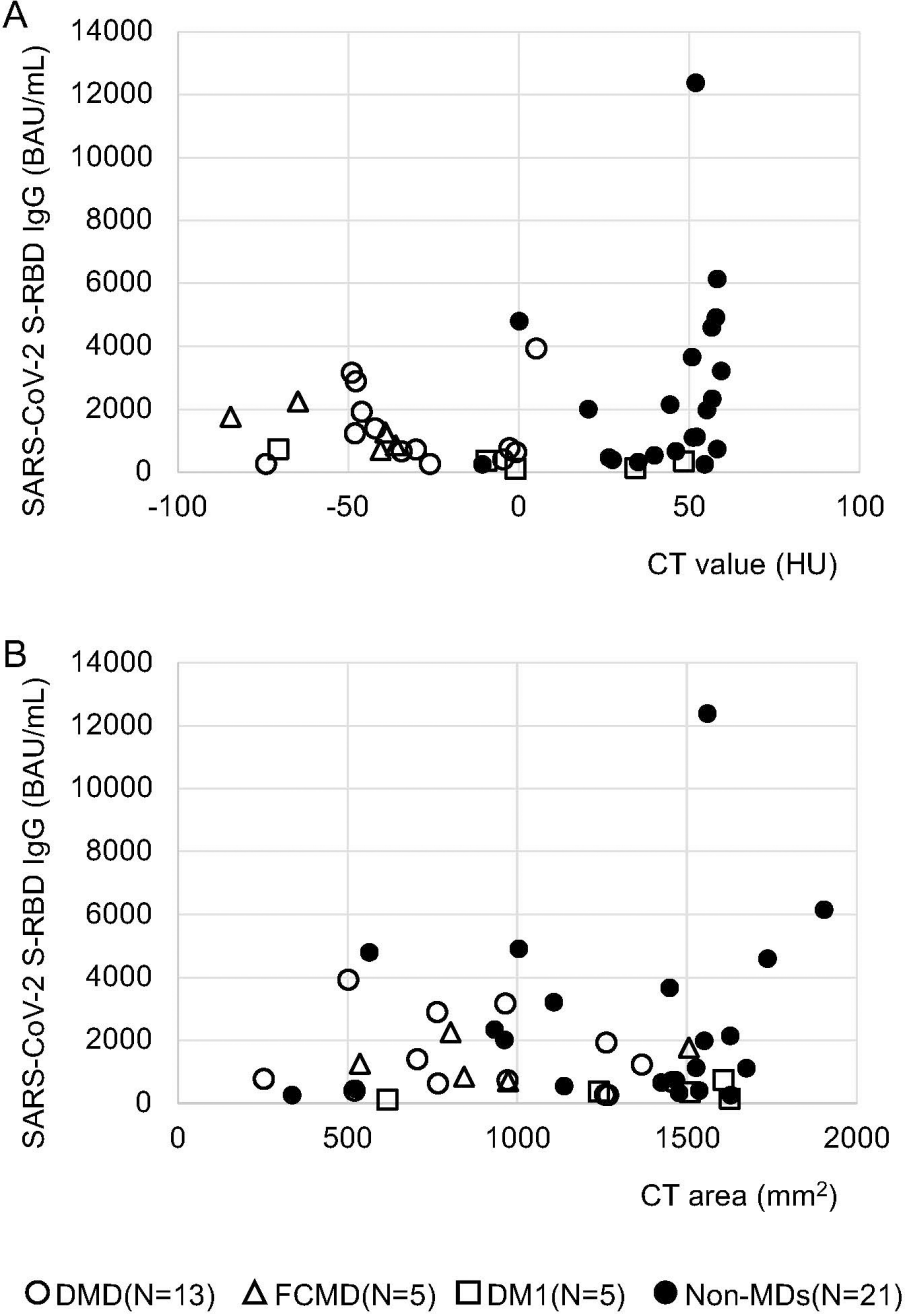
Correlations between anti-SARS-CoV-2 S-RBD IgG antibody levels and mean CT values, as well as areas of the deltoid muscle from CT images during the first month after the second vaccination. **(A)** Comparison of mean CT value ≤ 0 HU (n = 21) and mean CT value > 0 HU (n = 23); no significant difference was observed, but patients with mean CT values ≤ 0 HU had a low tendency of antibody response with intramuscular SARS-CoV-2 mRNA vaccines one month after the second vaccination. **(B)** Comparison of the area ≤ 1000 mm^2^ (n = 18) and the area > 1000 mm^2^ (n = 26); no significant difference was observed.

Similarly, a difference between patients with a mean CT value of zero HU and under for the deltoid muscle (mean 1,080.5 BAU/mL) and those with a mean CT value greater than zero HU (mean 2,527.0 BAU/mL) was observed at one month following the second vaccination with *p*-values of 0.09 (**Table 1**; **Figures 1**, **2A**). No differences were observed on the first day after the second vaccination, or three- and six-months post vaccination (*P* = 0.308, 0.178, and 0.426, respectively) (**Table 1**). In patients with and without MDs, the respective median of mean CT values of the deltoid muscle were -36.1 and 51.2 HU, and the respective mean of mean CT values of the deltoid muscle were -28.9 and 42.6 HU (**Figure 2A**).

Conversely, when we compared the area of the deltoid muscle of 1000 mm^2^ and under with those over the area of 1000 mm^2^, no differences were observed over time, i.e., on the first day of the second vaccination and one, three, and six months after the second vaccination (*p* = 0.15, 0.6, 0.29, and 0.17, respectively; **Table 1**; **Figures 1**, **2B**). In patients with and without MDs, the respective median CT areas of the deltoid muscle were 971.2 and 1468.4 mm^2^, and the respective mean CT values of the deltoid muscle were 1014 and 1341.3 mm^2^ (**Figure 2B**).

## Discussion

Our previous study showed that the immunogenicity of intramuscular SARS-CoV-2 mRNA vaccination in patients with MDs were lower than that of patients without MDs. We believed that MDs may be a significant key contributor in predicting the antibody response to intramuscular SARS-CoV-2 mRNA vaccines (3). However, the mechanism for the lower antibody response to intramuscular SARS-CoV-2 mRNA vaccination in patients with MDs remained unknown. In the current study, we indicated that the mean CT values of the deltoid muscle can account for this mechanism because a comparison of the immunogenicity of intramuscular SARS-CoV-2 mRNA vaccination between patients with and without MDs showed a similar tendency with that between mean CT values ≤ 0 HU and > 0 HU. On the basis of these findings, we posit that the quality of the muscle might affect the immunogenicity of intramuscular SARS-CoV-2 mRNA vaccination in patients with MDs.

Kuru et al. (10) indicated that chronological changes in the histograms of CT values were correlated with disease progression in proportion to the degree of loss of muscle fibers and the replacement of fatty tissues; this finding is consistent with that of the current study, namely, patients with MDs have lower CT values than patients without MDs. We could not evaluate the histological effects for the antibody response to intramuscular SARS-CoV-2 mRNA vaccines because it is difficult to distinguish muscle fiber from connective tissue owing to their similar CT values (10). However, we can at least describe that the antibody response of intramuscular SARS-CoV-2 mRNA vaccines in patients with MDs may be affected by the tissue characterization of the deltoid muscle at the injection site. In addition, data on a potentially sufficient humoral immune response when administering BNT162b1 subcutaneously were also reported (13). We believe that these findings support the association of the antibody response of intramuscular mRNA vaccines and the pathological condition of patients with MDs.

Three study groups reported that intramuscular SARS-CoV-2 mRNA vaccination resulted in a comfortable IgG antibody response in patients with MDs (14–16). Demeonbreun et al. (14) described that the presence of a normal immune response to intramuscular mRNA vaccination in patients with MDs indicated that non-muscle components significantly influence the antibody response. Iwayama et al. (15) described that intramuscular SARS-CoV-2 mRNA vaccination might increase antibody levels sufficiently via preserved vascular tissue, which allows the injected drug to reach the systemic circulation quickly even if intramuscular SARS-CoV-2 mRNA vaccines are administered to fat-replaced muscle. Saito et al. (16) reported that ambulatory status and DM1 (in addition to age) affected the low immunogenicity of intramuscular SARS-CoV-2 mRNA vaccination in patients with MDs. They mentioned that immobility and the resulting decrease in blood flow, which reduces the ability of mRNA vaccines to induce an immune response, should be considered because rich blood flow allows for the efficient processing of antigens (17, 18). Our studies indicated that the antibody response of intramuscular SARS-CoV-2 mRNA vaccines were not affected by ambulatory status, and anti-SARS-CoV-2 S-RBD IgG levels in patients with DM1 showed low tendency. Low levels of serum IgG and abnormalities in cellular and humoral immunity in patients with DM1 have also been reported previously (19, 20), but the mechanisms remain unclear. Taken together, we speculate that pathological condition in MDs might strongly affect the immunogenicity of intramuscular SARS-CoV-2 mRNA vaccination in patients with MDs.

This study has several limitations. First, the study size was small, and no significant differences were confirmed. The immunogenicity of intramuscular SARS-CoV-2 mRNA vaccination in patients with MDs, particularly those in the MD subgroups, needs to be further investigated in a study with a larger number of cases with MDs. We speculate that disease types and pathogenesis in MDs may be important contributing factors in predicting antibody response; therefore, further studies will lead to improvements in the management of MDs. Second, the evaluation method used to assess muscles in this study was only CT imaging. We could not use several imaging techniques including MRI and dual-energy X-ray absorptiometry (DXA) (21). However, CT imaging was thought to be best because MRI and DXA have difficulty in identifying the deltoid muscle and cause contamination of artifacts. Third, as previously mentioned (3), only total IgG antibody levels against the SARS-CoV-2 S-RBD were measured in the current study. Other studies have provided not only an assessment of the humoral immune response but also combined analyses of humoral and cellular immunity. In addition, our studies have shown the association of immunogenicity with SARS-CoV-2 vaccination in patients with MDs by using only an intramuscular mRNA vaccine. Therefore, we believe that the SARS-CoV-2 immunization of patients with MDs requires extensive research.

## Conclusion

We reported the association of the immunogenicity of intramuscular SARS-CoV-2 mRNA vaccination with CT muscle images in patients with or without MDs. Disease type and MD pathogenesis may be key contributors in predicting the antibody response to intramuscular SARS-CoV-2 mRNA vaccine. However, SARS-CoV-2 immunization in patients with MDs still require extensive research.

## Data Availability

The original contributions presented in the study are included in the article/supplementary material. Further inquiries can be directed to the corresponding author.
